# Data on the porphyrin effect and influence of dopant ions on *Thaumatococcus daniellii* dye as sensitizer in dye-sensitized solar cells

**DOI:** 10.1016/j.dib.2018.09.017

**Published:** 2018-09-12

**Authors:** Temitope Jolaolu Abodunrin, Adenike Omotunde Boyo, Mojisola Rachael Usikalu

**Affiliations:** a Department of Physics, Covenant University, P.M.B. 1023, Ota, Nigeria; b Department of Physics, Lagos State University, P.M.B. 0001, Ojo, Nigeria

**Keywords:** Organic dye, Porphyrin, Efficiency, UV/VIS spectroscopy

## Abstract

In this work, data on the effect of porphyrin characteristic of UV/VIS absorption of *Thaumatococcus daniellii* (*T. daniellii*) dye-sensitized solar cells sensitized with different electrolytes were presented. The influence of dopants from 1 g/100 ml electrolyte: distilled water and applied time difference of 3 min is observed on the photovoltaic characteristics and performance of the deposited thin film. The output efficiency and incident photon to conversion efficiency of *T. daniellii* dye-sensitized solar cells was acquired and could be further used as a model for designing dye-sensitized solar models as substitute for silicon solar cells

**Specifications table**

**Table:** 

Subject area	*Materials Science*
More specific subject area	*Dye-sensitized solar cells*
Type of data	*Table, image*
How data was acquired	The photovoltaic characterization took place under 1.5 standard condition of air mass. The volume of four different electrolytes was constant and introduction of the ions was by use of a 21 G × 1.5 in. hypodermic needle in-between two transparent conducting slides. Doctor blade application of photoanode was employed with high temperature sintering at 450 °C in an autoclave. The thin film depth was measured using a profilometer. The photovoltaic value was obtained from parallel connection of each doped dye-sensitized solar cell with a variable resistor and a digital multimeter.
Data format	Raw, Analyzed
Experimental factors	The weight of *T. daniellii* dye was measured appropriately and volume of electrolyte in distilled water was obtained before the injection was done and required data acquired.
Experimental features	The thin film depositions were performed on an active area of 6.25 cm^2^ and temperature of 38 °C. The effect of ionic dopant difference on the photovoltaic properties of the *T. daniellii solar cells* and porphyrin characteristic absorbance was acquired, at a current density interval of 3 min for determining the potential difference for different loads. The irradiance of Ota in Nigeria as it influences the crop of sunlight harvested and roughness factor of the dye was put into consideration.
Data source location	Renewable Energy Research Laboratory, Department of Physics, Covenant University, Ota, Ogun State, Nigeria
Data accessibility	Data are available within this article

**Value of the data**

• The given data will demonstrate to authors in materials science with specialization in dye-sensitized solar cells the correlation between influence of ionic dopant on reaction kinematics and photovoltaic performance of *Thaumatococcus daniellii* (*T. daniellii*) dye-sensitized solar cells.
• The data obtained for the mercury ion dopant can be used as material for theoretical simulation for higher efficiency dye-sensitized solar cells.
• The data can be used to illustrate the relationship between UV/VIS absorbance characteristic as it affects the photovoltaic characterization of *T. daniellii* dye sensitized solar cells.
• The data could be used for investigating the progression of porphyrin efficiency in dye-sensitized solar cells relative to that of a solar simulator.
• The data obtained can be used in investigating the porphyrin behaviour of *T. daniellii* dye in reaction to different dopants in an electrolyte, dye cocktails with synthesized dyes or other N3 or N719 dyes relating to their kinematics and photovoltaic characteristics.

## 1 Data

**Fig. 1:**
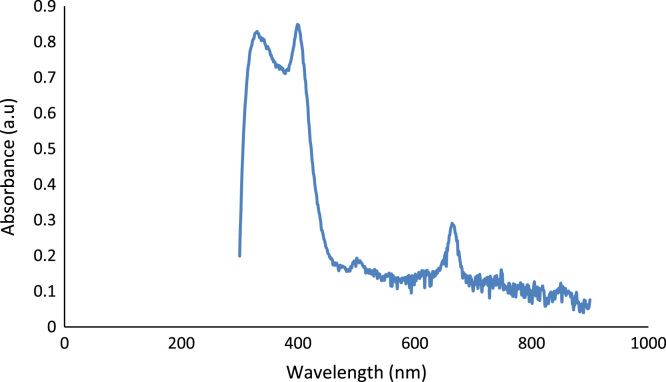
UV/VIS of *T. daniellii* dye.

**Fig. 2:**
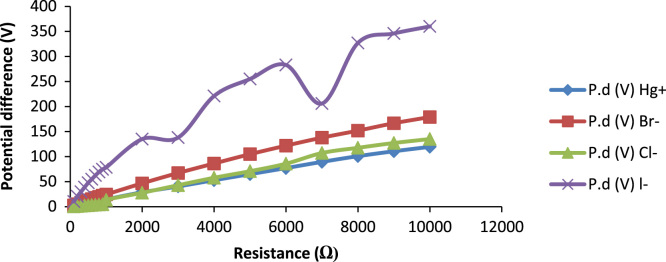
Variation of potential difference and effect of dopants on resistance.

**Fig. 3:**
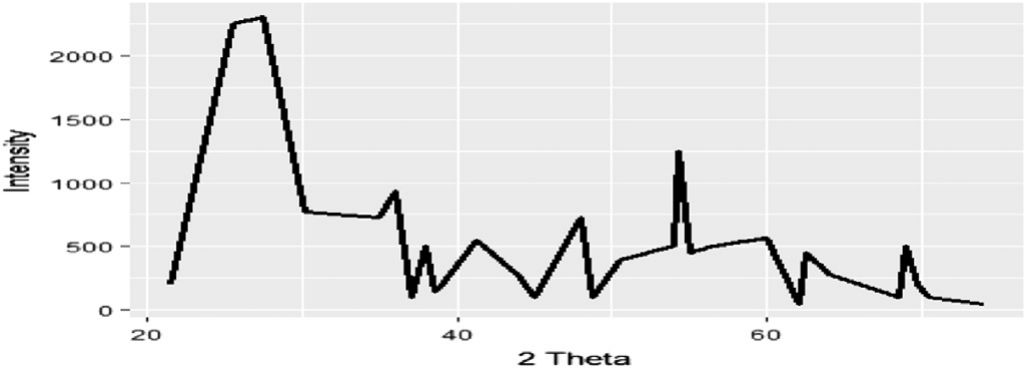
Theoretical simulation of efficient charge transport pathway.

The data generated from the experiment are on variation of ion dopants in dye-sensitized solar cells. The ionic deposition was performed at 8.5 mm width depth and a temperature of 38 °C. The data acquired from UV/VIS spectroscopic analysis of *Thaumatococcus daniellii* (*T. daniellii*) is presented in Fig. 1. The absorbance of *T. daniellii* reveals strong absorbance in soret and Q bands respectively. The significance of this is that the crop of sunlight harvested is larger across the electromagnetic spectrum which agrees with other research work [1], [2]. The influence of different chromophores on absorbance were considered as shown in Table 1 and each photovoltaic result is compared with others as representative data for better precision as shown in Fig. 2. This enquiry was considered necessary because of the low output performance of liquid electrolyte dye-sensitized solar cells to obtain the required data for theoretical simulation presented in Fig. 3.

**Table 1:** Data showing Fourier transform infrared (FTIR) of *T. daniellii* dye.

	Absorption peak (cm)^−1^	Type of bond	Assignment
**1.**	505.37; 584.45	Bromoalkanes	Medium to strong appearance
**2.**	684.75; 738.76; 837.13	C–X	Weak to medium appearance
**3.**	790.84; 833.28	S–OR	Strong appearance
**4.**	970.23; 1041.6; 1097.53; 1192.05; 1240.27	P–OR esters and P=O	Strong appearance
**5.**	1300.07; 1377.22; 1452.45; 1637.62; 1741.78; 2362.88; 2729.37; 2854.74; 2926.11	Carboxylic acids and derivatives	Strong appearance
**6.**	3439.19	Amines in dilute solution	Weak appearance

## 2 Experimental design, materials and methods

**Fig. 4:**
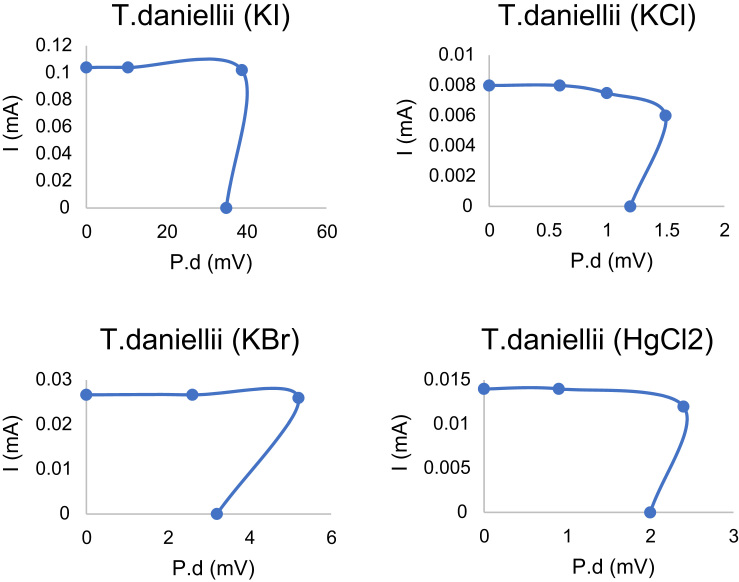
*I*–*V* plots of *T. daniellii* with different electrolytic dopants.

*T. daniellii dye* was extracted from 4000 ml of its methanolic solution with 200 g of *T. daniellii* leaves commercially bought from a vegetable market in Nigeria. Phytochemical screening revealed a chromophore rich compound in carbohydrate, tannin, saponin, flavonoid, steroid, alkaloid and cardiac glycoside. The pathway used for charge transport is the porphyrin-flavonolic pathway in which the flavonoid attaches itself to *T. daniellii* moiety to elongate the molecule and aid absorption of photons of light within the visible spectrum as illustrated by Table 1 [2]. Indium doped tin oxide conducting glass of dimension (2.5 cm × 2.5 cm × 0.01 cm) was sourced and sectioned into (1.5 cm × 1.20 cm × 0.01 cm) as active area of exposure to sunlight served as the photoanode. The photoanode comprised of a uniform blend of TiO_2_ Degussa and conc. HNO_3_ applied on the active area. The counter electrode made by soot coated epitaxial layers on indium doped tin oxide from a naked Bunsen flame in a simulated vacuum. The slides were of surface resistivity 10 Ω/m^2^. Initial surface preparation was performed by demarcating the ITO using masking tape on an active surface area of 3.14 m^2^ as described in our previous studies [3], [4], [5]. Each 0.1 g of dye extract was grown on the photoanode by inserting the ITO vertically in the dye solution. Thus, the dye soaked into the TiO_2_ framework via capillary action. The set-up was allowed to dry before the two electrodes were coupled together with binder clips. 1 ml of potassium bromate, potassium chloride, mercury chloride and potassium iodide were dissolved in deionized water to give the aqueous electrolyte solution [6], [7], [8], [9], [10], [11]. The doped specimens were obtained from injecting the middle of the sandwich of electrodes with the resulting electrolytic solution. The result obtained from the photovoltaic characterization of the samples is shown in Table 2 as obtained from 3650 digital multimeter [12], [13], [14], [15]. The duration of obtaining the photoelectric values was 3 min as described by [16], [17], [18], [19]. X-ray diffraction (XRD) micrograph of *T. daniellii* was modelled with gg plots of Octave software to identify the best conduit for charge transport. The result of modelling is given by the efficient path shown in Fig. 3. The software program used to obtain the plot is accessible from the Appendix A. *I*–*V* plot of *T. daniellii* dye-sensitized solar cells is presented in Table 3. The photovoltaic characterization parameter for *T. daniellii* under the influence of dopants is as shown on Table 4 and illustrated by Fig. 4. The choice of electrolyte is due to a preliminary study from our previous work [20], [21]. The efficiency (*η*) and incident photon to conversion efficiency of *T. daniellii* DSCs were determined from Eqs. (1), (2) respectively. This expresses the ratio of power output obtained from the DSC to the power input and quantum efficiency respectively expressed as a percentage.

(1) $$\eta =\frac{V_{OC}I_{SC}ff}{P_{in}}$$

(2) $$IPCE=\frac{I_{SC}\times 1240}{P\times \lambda }\times 100$$

**Table 2:** Data showing potential difference of *T. daniellii* DSCs connected across various loads for different ion dopants in 3 min.

Resistance (Ω)	P.d (mV) Hg^+^	Br^−^	Cl^−^	l^−^
**100**	0.9	2.6	0.6	10.4
**200**	2.4	5.2	1.2	20.8
**300**	3.8	8.0	1.8	29.9
**400**	5.6	10.4	2.4	38.9
**500**	7.1	12.8	2.9	47.8
**600**	8.7	15.3	3.4	55.0
**700**	10.2	17.9	3.9	62.5
**800**	11.8	20.0	4.3	69.4
**900**	13.3	22.2	4.6	74.8
**1000**	14.7	24.5	13.5	78.5
**2000**	28.8	46.7	27.8	135.0
**3000**	40.5	67.3	42.9	138.1
**4000**	52.9	86.3	57.8	221.0
**5000**	65.0	104.9	70.9	255.0
**6000**	77.1	121.9	85.9	283.0
**7000**	89.5	137.9	107.2	206.0
**8000**	100.9	151.9	117.6	327.0
**9000**	110.7	166.7	127.2	346.0
**10,000**	119.5	179.0	135.1	360.0

**Table 3:** Data showing *T. daniellii׳s* current-voltage parameters for four different electrolytes.

Resistance (Ω)	HgCl_2_*I* (mA)	P.d (mV)	KBr *I* (mA)	P.d (mV)	KCl *I* (mA)	P.d (mV)	KI *I* (mA)	P.d (mV)
100	0.0	0.0090	0.0	0.0260	0.0	0.0000	10.4	0.1040
200	0.9	0.0090	2.6	0.0260	0.6	0.0060	20.8	0.1040
300	2.4	0.0120	5.2	0.0267	1.2	0.0060	29.9	0.0997
400	3.8	0.0130	8.0	0.0260	1.8	0.0060	38.8	0.0970
500	5.6	0.0140	10.4	0.0256	2.4	0.0060	47.8	0.0956
600	7.1	0.0142	12.8	0.0255	2.9	0.0058	55.0	0.0917
700	8.7	0.0145	15.3	0.0256	3.4	0.0057	62.5	0.0893
800	10.2	0.0146	17.9	0.0250	3.9	0.0056	69.4	0.0868
900	11.8	0.0148	20.0	0.0247	4.3	0.0054	74.8	0.0831
1000	13.3	0.0148	22.2	0.0245	4.6	0.0051	78.5	0.0785
2000	14.7	0.0147	24.5	0.0234	13.5	0.0135	135.0	0.0675
3000	28.8	0.0144	46.7	0.0224	27.8	0.0139	138.1	0.0460
4000	40.5	0.0135	67.3	0.0216	42.9	0.0143	221.0	0.0553
5000	52.9	0.0132	86.3	0.0210	57.8	0.0145	255.0	0.0510
6000	65.0	0.0130	104.9	0.0203	70.9	0.0142	283.0	0.0472
7000	77.1	0.0129	121.9	0.0197	85.9	0.0143	206.0	0.0294
8000	89.5	0.0128	137.9	0.0190	107.2	0.0153	327.0	0.0409
9000	100.9	0.0126	151.9	0.0185	117.6	0.0147	346.0	0.0384
10,000	110.7	0.0123	166.7	0.0179	166.7	0.0179	360.0	0.0360
*V_oc_*	119.5	0.0120	179	0	179	0	123.0	0.0000

**Table 4:** Data showing the influence of ion dopants on *T. daniellii׳s* photovoltaic parameters from four different electrolytes.

Electrolyte	*I_sc_* (mA)	*V_oc_* (mV)	Fill factor (%)	*P_max_* (W)	*ƞ* (%)	IPCE(%) × 10^−3^
KCl	0.006	120.8	2.5	1.819	0.01	0.010
HgCl_2_	0.009	97.9	1.5	1.362	0.43	0.010
KBr	0.026	123.8	1.0	3.083	0.01	0.010
KI	0.102	302.0	0.3	9.110	0.03	0.003

## Appendix A

**R Octave – gg plot**.

R-Shining:

#populate data on excel.

#log data into R studio.

#Tools-Global-Appearance.

#xy plot (intensity~2theta, data= XRD).

#grid on.

x label (‘theta’).

y label (‘f(x)=e^ ∫).

#help(log).

Prod().
