# Supplementary material for: Data on the porphyrin effect and influence of dopant ions on Thaumatococcus daniellii dye as sensitizer in dye-sensitized solar cells
Source: Data Brief. 2018 Sep 12;20:2020–6. doi: 10.1016/j.dib.2018.09.017 (PMC6172415; doi:10.1016/j.dib.2018.09.017)
Supplement: Supplementary file 1 — Supplementary material [file mmc1.docx]

**COVER LETTER**

The authors of the manuscript titled: **Data on the porphyrin effect and influence of dopant ions on *Thaumatococcus daniellii* dye as sensitizer in dye-sensitized solar cells** hereby declare that, this is an original research work. It is not undergoing review currently and has never been published in this form in any journal.

The authors also declare that, there is no conflict of interest whatsoever to the publication of this article. The publication was written for generating scientific impact to a global network of researchers which DIB journal reaches through their quality publications.

Yours Sincerely,

Temitope Abodunrin
